# The impact of the United States’ distancing from the UN and withholding WHO funding on maternal and child health and nutrition in the Latin America & Caribbean region

**DOI:** 10.1186/s12939-026-02785-3

**Published:** 2026-03-12

**Authors:** Mónica Ancira-Moreno, Rafael Pérez-Escamilla, Ana Lorena Ruano, Alejandra Cantoral, Fernanda Cobo Armijo, Graciela Teruel Belismelis, Sonia Hernández-Cordero

**Affiliations:** 1 Health Department, Universidad Iberoamericana, Mexico City, Mexico; 2 Observatorio Materno Infantil (OMI), Universidad Iberoamericana, Mexico City, Mexico; 3 Department of Social and Behavioral Sciences, Yale School of Public Health, New Haven, CT USA; 4 Center for International Health, Department of Global Public Health and Primary Care, University of Bergen, Bergen, Norway; 5 Department of Law, Universidad Iberoamericana, Mexico City, Mexico; 6 Social Sciences Division, Universidad Iberoamericana, Ciudad de México, México; 7 Research Center for Equitable Development EQUIDE, Universidad Iberoamericana, Mexico City, Mexico; 8 Universidad Iberoamericana, Prolongación, Av. P.º de La Reforma 880, Santa Fe, Álvaro Obregón, Ciudad de México, 01219 México

**Keywords:** Maternal health, Child nutrition, Latin America and the Caribbean (LAC), World Health Organization (WHO), United Nations funding, Health systems strengthening, Health financing

## Abstract

This commentary examines the serious implications of the United States’ (US) decision to withdraw funding from the World Health Organization (WHO) and the United Nations (UN) system for maternal and child health and nutrition in Latin America and the Caribbean (LAC). The region has made significant progress in reducing maternal and infant mortality through coordinated efforts led by WHO, PAHO, and other UN agencies, as an example, infant mortality decrease in LAC by 67% between 1990 and 2015. However, many countries, particularly low-income countries and small island nations, remain highly dependent on international funding and technical support for essential services, such as maintaining immunization coverage and reducing maternal and infant mortality. Importantly, the impacts on the health of LAC due to the US departure from WHO can be devastating, by contributing to the weakening of epidemiological surveillance, the reduction of the capacity to respond to disasters and other public health emergencies, and affecting services for vulnerable populations, such as women and children. The US, historically the largest WHO donor (22% of its budget), has supported essential programs such as vaccination, neonatal care, nutrition, and health services for migrant populations. This major disruption in funds is especially concerning for vulnerable communities, and will likely increase the risk of disease resurgence. It will also weaken regional preparedness for future pandemics. This commentary calls for urgent action from LAC governments to safeguard public health. Recommended strategies include diversifying funding through earmarked taxes, social impact bonds, and public-private partnerships, and increasing domestic health investment. Allocating at least 2% of GDP to maternal and child health could help mitigate the impact of global policy shifts and reduce internal inequities, ensuring continued progress in the region.

## Background

Tackling maternal and child health and nutrition challenges demand a coordinated response. Global leadership, normative guidelines, and support from the World Health Organization (WHO) and United Nations (UN) agencies have been key to strengthening health systems and improving outcomes in Latin America & the Caribbean (LAC). The recent decision by the United States (US) to withdraw funding from the UN in general, and the WHO in particular, marks a pivotal moment in global health governance [1]. As the largest contributor to these global institutions, the US has historically played a crucial role in financing initiatives that address infectious diseases, maternal and child health, and emergency responses [2]. This policy shift threatens to destabilize public health in Latin America, a region already burdened by health inequities, emerging epidemics, climate-related health crises, and increasing migration, which further strain fragile healthcare systems and essential services^2–3^. As reported by WHO, decades of progress will be reversed due to loss of health workers, broken supply-chains for essential medicines, and interruption of vital health and social protection programs [3].

## Main text

### The financial and structural impact of this decision

The WHO relies on assessed and voluntary contributions, with the US accounting for 22% of its budget [4]. The sudden reduction of these funds directly jeopardizes programs supported by the Pan American Health Organization (PAHO), which have been instrumental in improving maternal and neonatal care and strengthening health systems in Latin America [5]. The Global Polio Eradication Initiative, for instance, receives substantial financial support from the WHO, and a financial shortfall of this scale could lead to polio resurgence across regions. In addition, context-specific interventions, such as those addressing the regional migration crisis led by the United Nations Children’s Fund (UNICEF), the International Organization for Migration (IOM), and the United Nations High Commissioner for Refugees (UNHCR), are at risk due to funding withdrawals. In some cases, this has already led to a complete halt to essential healthcare services that governments cannot provide for these vulnerable populations [4].

### Consequences for maternal and child health

The LAC region has made significant strides in reducing maternal and infant mortality, largely through coordination across UN agencies with strong leadership from WHO and PAHO [5]. However, many health systems in the region remain fragile, characterized in the context of public insecurity and a weak rule of law, economic and significant migratory pressures, territorial inequality and insufficient investment in health (< 2% of GDP) [6, 7], with high dependence on international technical support and funding for primary healthcare initiatives.

The withdrawal of US funds may lead to setbacks in progress, through the disruptions in vaccine procurement and distribution, causing the reappearance of cases and even national outbreaks of pathogens that were recently under control [8]. The COVID-19 pandemic highlighted how vulnerable health systems were even before the US withdrawal from the WHO, illustrating how a lack of support for one region can easily translate into a risk that local outbreaks will rapidly become a pandemic. Likewise, nutrition programs and emergency obstetric care disproportionately affect marginalized and vulnerable communities [7]. Without sustained funding, progress in reducing neonatal mortality and improving childhood nutrition could stagnate or reverse.

### The risk of infectious disease reemergence

The COVID-19 pandemic demonstrated the need for a robust, coordinated, international response. The WHO-led initiatives, such as the COVAX initiative, was designed to provide vaccines to low- and middle-income countries, to prevent a more devastating health crisis in the region [8]. The withdrawal of US contributions will indeed weaken the WHO’s capacity to manage future pandemics, mitigate antimicrobial resistance, and respond to endemic diseases like dengue and Zika virus infection, which disproportionately affect LAC^9^.

### The path forward: call for global solidarity

At a time when global health threats are increasingly interconnected, unilateral financial disengagement from multilateral organizations threatens collective security. Governments from LAC, alongside regional health institutions and philanthropic organizations, must explore alternative, diversified funding mechanisms such as the creation of dedicated funds for medicines or earmarked taxes to protect public health, such as taxes on sugary drinks applied in more than 20 countries in Latin America and the Caribbean [9, 10]. Additionally, social impact bonds and research and development capital investment through public-private partnerships are used to bridge the financial gap. Strengthening regional collaborations, such as implementing front-of-pack labeling on packaged food products in the LAC region, to reduce consumption of the highly harmful ultra-processed foods [11]. Increasing domestic investment in health systems and leveraging partnerships with the European Union and emerging global health donors will be essential. It is important to note that PAHO estimates that the countries that have made the most progress toward universal coverage have public health spending levels of at least 6% of their gross domestic product (GDP) [10].

In this context, it is key to call on the Governments of the LAC region to allocate at least 2% of GDP to maternal and child health to mitigate the impact of sweeping policy changes in the United States [12]. Furthermore, increasing overall public health spending in LAC by approximately 2 additional percentage points of GDP is now even more justified, given both the elimination of US funding for global health and the persistent gap relative to the 6% of GDP minimum threshold recommended by PAHO/WHO [13]. These investments need to be made in a thoughtful way, as the ultimate goals should be to reduce chronic funding deficits, strengthen primary care, advance toward universal coverage, and reduce inequalities in the region through sustainable, long-term programs [14].

## Conclusion

The US withdrawal of funds from the UN and exit from the WHO represents an urgent challenge to global health, with LAC among the most affected regions. Urgent action is needed—leaders must secure funding and protect progress in maternal-child health and nutrition. Reversing these gains is not an option. Protecting public health requires a unified commitment to combating infectious diseases, strengthening health systems, and upholding collective health governance.

## Data Availability

Not applicable as we did not collect any data.

## Abbreviations

US: United States
WHO: World Health Organization
UN: United Nations
LAC: Latin America and the Caribbean
PAHO: Pan American Health Organization
UNICEF: United Nations Children’s Fund
IOM: International Organization for Migration
UNHCR: United Nations High Commissioner for Refugees
GDP: Gross Domestic Product
